# The Proresolving Lipid Mediator Maresin1 Alleviates Experimental Pancreatitis via Switching Macrophage Polarization

**DOI:** 10.1155/2021/6680456

**Published:** 2021-03-09

**Authors:** Yingying Lu, Guotao Lu, Lin Gao, Qingtian Zhu, Jing Xue, Jingzhu Zhang, Xiaojie Ma, Nan Ma, Qi Yang, Jie Dong, Weijuan Gong, Weiqin Li, Zhihui Tong

**Affiliations:** ^1^Department of Surgical Intensive Care Unit (SICU), Department of General Surgery, Jinling Hospital, Medical School of Southeast University, No. 305 Zhongshan East Road, Nanjing, 210002 Jiangsu, China; ^2^Pancreatic Center, Department of Gastroenterology, Affiliated Hospital of Yangzhou University, No. 368 Hanjiang Media Road, Yangzhou, 225000 Jiangsu, China; ^3^Surgical Intensive Care Unit (SICU), Department of General Surgery, Jinling Hospital, Medical School of Nanjing University, No. 305 Zhongshan East Road, Nanjing, 210002 Jiangsu, China; ^4^State Key Laboratory of Oncogenes and Related Genes, Stem Cell Research Center, Ren Ji Hospital, School of Medicine, Shanghai Jiao Tong University, Shanghai 200127, China

## Abstract

**Method:**

Repeated caerulein injection was used to induce AP and chronic pancreatitis (CP) models in mice. The histopathological and serological changes were examined for evaluating the severity of the AP model, and flow cytometry was used for detecting macrophage phagocytosis and phenotype. Meanwhile, clodronate liposomes were used for macrophage depletion in mice. Finally, the CP model was adopted to further observe the protective effect of MaR1.

**Result:**

MaR1 administration manifested the improved histopathological changes and the lower serum levels of amylase and lipase. However, MaR1 played no protective role in the pancreatic acinar cell line *in vitro*. It obviously reduced the macrophage infiltration in the injured pancreas, especially M1-type macrophages. After macrophage clearance, MaR1 showed no further protection *in vivo*. This study also demonstrated that MaR1 could alleviate fibrosis to limit AP progression in the CP model.

**Conclusion:**

Our data suggests that MaR1 was a therapeutic and preventive target for AP in mice, likely operating through its effects on decreased macrophage infiltration and phenotype switch.

## 1. Introduction

Acute pancreatitis (AP) is one of the common acute abdominal fatal diseases. Severe AP (SAP) accounts for about 15%-20% of patients with AP and develops approximately 30% mortality [1, 2]. Activated innate immune cells increase the severity of AP. The prognosis of AP is related to an excessive inflammatory response. In this process, deregulated immune cells mediate the inflammatory cascade, leading to systemic inflammatory response syndrome and multiple organ failure [3]. However, no cure is available for AP, especially SAP patients with persistent organ failure.

Omega-3 polyunsaturated fatty acids (*ω*-3 FAs) mainly include docosahexaenoic acid (DHA) and eicosapentaenoic acid. *ω*-3 FA/DHA plays an indisputable role in hyperlipidemia and cardiovascular diseases [4, 5]. Studies involving humans as participants indicated that the use of enteral nutrition enriched with *ω*-3 FAs for treating AP might be beneficial to the time of jejunal feeding and hospital stay [6]. A meta-analysis revealed that administering *ω*-3 FAs might be beneficial for decreasing mortality, infection-related complications, and length of hospital stay in AP, especially when used parenterally [7]. Previous clinical data indicated that *ω*-3 FA-supplemented parenteral nutrition could decrease hyperinflammatory response and improve immune function of patients with SAP [8, 9].

Inflammation can be divided into three phases: inflammation, resolution, and post-resolution [10]. Specialized proresolving mediators (SPMs), including resolvins, protectins, lipoxins, and maresins, play a key role in resolution and postresolution [11, 12]. Inflammation is a protective response in maintaining homeostasis. However, excessive inflammation may lead to injury in normal tissues and finally develop into chronic diseases [2, 13]. SPMs provide a new avenue for inflammation; they prevent inflammation from spreading and halt the transition from acute to chronic [14, 15].

Maresins are newly described macrophage-derived mediators of inflammation resolution; they are one of the metabolites from *ω*-3 FAs and biosynthesized via 12-lipoxygenase [14, 16, 17]. Maresin1 (7,14-dihydroxyd-ocosa-4Z, 8Z,10,12,16Z,19Z-hexaenoic acid, MaR1) has been shown to be a potent mediator to inhibit neutrophil infiltration, promote macrophage efferocytosis, and enhance tissue regeneration in acute inflammation [18–20]. MaR1 exerts protective effects in murine models of colitis and sepsis [21, 22]. Recently, it has been reported that MaR1 protected mice from nonalcoholic fatty liver disease in a ROR*α*-dependent manner [23]. Several studies reported the protective effects of MaR1 on AP without clarifying its specific target cells [24, 25].

Compared with other SPMs, the effect of MaR1 on AP remains unknown. The purpose of this study was to verify the hypothesis of whether MaR1 could protect against AP, as well as exploring the possible underlying mechanism.

## 2. Materials and Methods

### 2.1. Animals and Reagents

ICR male mice (aged 8 weeks), weighing 28-30 g, were purchased from the Yangzhou University Model Animal Center (Yangzhou, China). Green fluorescent protein transgenic (GFP tg) mice were obtained from Prof. BJ Wu. All mice were housed under specific pathogen-free (SPF) conditions in an air-conditioned animal facility at 24°C on a 12 hours light/dark cycle. The Principles of Laboratory Animal Care (NIH publication no. 85Y23, revised 1996) were followed, and all experimental protocols were approved by the experimental animal ethics committee of Jinling Hospital affiliated to Medical School of Nanjing University (No. 20160905).

The murine pancreatic acinar 266-6 cell line was obtained from the American Type Culture Collection (ATCC, VA, USA). MaR1 was purchased from Cayman Chemical Company (MI, USA). Caerulein was obtained from AnaSpec Inc. (CA, USA). Cholecystokinin fragment 30-33 amide (CCK) and lipopolysaccharide (LPS) were purchased from Sigma-Aldrich (MO, USA); clodronate liposomes (CLs, from Vrije Universiteit Amsterdam) were purchased from Yeasen Biotech Co., Ltd. (Shanghai, China). The lipase kits were purchased from Nanjing Jiancheng Corp. (Nanjing, China), and the amylase kits were purchased from BioSino BioTechnology & Science Inc. (Beijing, China). Macrophage-stimulating factor (M-CSF) was purchased from MedChenExpress LLC. (Nanjing, USA). Anti-F4/80, anti-phospho-mixed lineage kinase domain like protein (p-MLKL), goat anti-rabbit, and rabbit anti-mouse secondary antibodies were purchased from Abcam (Cambridge, UK). Anti-receptor-interacting protein 3 (RIP3) antibody was purchased from Santa Cruz Biotechnology (CA, USA). All antibodies for flow cytometry were purchased from BioLegend (CA, USA). Dulbecco's modified Eagle's medium (DMEM), fetal bovine serum, penicillin, and streptomycin were obtained from Gibco (Thermo Fisher Scientific, MA, USA).

### 2.2. Induction of Experimental Pancreatitis

The mice were randomly assigned to five groups: control, caerulein, caerulein+0.2 ng/mice MaR1, caerulein+2 ng/mice MaR1, and caerulein+10 ng/mice MaR1. The AP model was induced by intraperitoneal (i.p.) injection of 50 *μ*g/kg caerulein every hour for 10 hours. Normal saline (NS, 0.9% NaCl) was given instead of caerulein in the control group. MaR1 or vehicle (0.9% NaCl) was injected intraperitoneally at 0 hour after the first caerulein injection. The mice were sacrificed 12 hours after the final injection.

Pancreatic macrophage depletion was in accordance with the instructions. Briefly, the mice were intraperitoneally administered 200 *μ*L of CLs on days 1 and 3. The control mice received the same volume of empty liposomes (phosphate-buffered saline, PBS). On the fifth day, the AP model was induced.

Chronic pancreatitis (CP) was induced by injecting of 50 *μ*g/kg caerulein once daily for a continued cycle of 5 days on and 2 days off, for a total of 4 weeks [23]. Seven days following the start of the caerulein injection, the mice were given either NS or MaR1 (2 ng/mice, 100 *μ*L daily for 5 days per week × 4 weeks) until sacrificed 5 weeks later.

### 2.3. Sample Collection and Analysis of Plasma Parameters

Blood samples were obtained from the tail veins of isoflurane-anesthetized mice 0, 6, and 12 hours after the first caerulein injection. The mice were anesthetized with sodium pentobarbital (50 mg/kg, i.p.) and sacrificed. Pancreatic tissues were taken and fixed with 4% paraformaldehyde in PBS (pH = 7.4) and embedded in paraffin.

The serum amylase and lipase activities were determined using amylase and lipase kits following the manufacturer's protocol.

### 2.4. Histological Examination

The paraffin sections of the pancreas and lung tissue were stained with hematoxylin and eosin (HE). Two investigators who were blinded to the experimental grouping scored the degree of pancreatic injury using light microscopy and evaluated the severity of edema, inflammation, and necrosis, as described previously [26].

### 2.5. Immunohistochemical Examination

The slices from paraffin-embedded pancreatic tissues were subjected to immunohistochemical (IHC) staining for RIP3, p-MLKL, and F4/80 detection. The slides were incubated overnight at 4°C in a humid chamber with an antibody against RIP3, p-MLKL and F4/80 (1 : 200 dilution) and then incubated with goat anti-rabbit secondary antibody (1 : 500 dilution) for 60 minutes as described previously [24]. The images were acquired using a microscope (IX73, Olympus, Tokyo, Japan).

### 2.6. Cell Cultures and Treatment

266-6 cells were cultured in DMEM supplemented with 10% fetal bovine serum, 100 U/mL penicillin, and 100 *μ*g/mL streptomycin in a humidified 5% CO_2_ incubator. Cholecystokinin analog CCK (8000 *μ*M) was applied to induce AP with or without MaR1 (250 nM, 500 nM, and 1000 nM). The cells were collected after 12 hours for further investigation.

### 2.7. Isolation of Pancreatic Leukocytes

MaR1 or vehicle was injected to treat the AP model. After 24 hours, the mice were killed, and the pancreas was removed carefully by trimming fat and mesentery. As mentioned in the literature [27], the pancreas was minced with scissors and then washed twice with buffer A (Hank's balanced salt solution +10% fetal calf serum). The tissue was resuspended in buffer A containing 2 mg/mL collagenase type IV and incubated in a shaker at 37°C for 15 minutes. The suspension was then vortexed at low speed for 20 seconds and centrifuged, and the cell pellet was resuspended in red blood cell lysis buffer for 5 minutes. The cells were spun down, washed three times with buffer A and used for marker staining.

### 2.8. Isolation of Primary Pancreatic Acinar Cells (PACs)

GFP tg mice emit green fluorescence spontaneously in the pancreas. PACs were isolated from GFP tg mice by collagenase digestion and then incubated in DMEM containing 10% fetal bovine serum at 37°C [28]. Bone marrow-derived macrophages (BMDMs) were coincubated with PACs, caerulein (5 *μ*M), and MaR1 (250 nM, 500 nM, and 1000 nM) for 6-8 hours.

### 2.9. Isolation of BMDMs and Cell Induction

Briefly, both ends of the femur and tibia were cut and flushed with a syringe filled with complete Roswell Park Memorial Institute (RPMI) 1640 containing 10% fetal bovine serum, 100 U/mL penicillin, 100 *μ*g/mL streptomycin, 1% N-2-hydroxyethylpiperazine-N-2-ethane sulfonic acid (HEPES, 1 M), and 0.05% *β*-hydroxy-1-ethanethiol to extrude BM cells into a sterile Petri dish. After gentle resuspension and centrifugation, BM cells were cultured using 20 ng/mL M-CSF in complete RPMI. On days 2, 4, and 6, the medium was half-replaced with a fresh batch containing the CSF-conditioned medium as earlier. The cells were ready for use on day 7 [28].

BMDMs were coincubated with caerulein-stimulated acini for 6 hours. After removing acini, BMDMs were collected and stained with fluorochrome-conjugated antibody: PE/Cy7-F4/80 for flow cytometry.

### 2.10. Flow Cytometry

Pyridine iodide (PI, 1 *μ*mol/L) was used to detect plasma membrane rupture characteristic of necrosis. After loading, 266-6 cells were washed and resuspended in Ca^2+^-free buffer.

For surface staining, pancreatic leukocytes were stained with the following fluorochrome-conjugated antibodies: FITC-CD86, PE/Cy7-CD45.2, APC/Cy7-CD11b, and Percp/Cy5.5-F4/80. For intracellular tumor necrosis factor *α* (TNF-*α*) staining, the cells were cultured in DMEM complete medium and stimulated with LPS (100 ng/mL) and brefeldin A (10 *μ*g/mL) for 4 hours at 37°C. The cells were washed and stained with surface markers. The cells were then fixed and permeabilized. PE-TNF-*α* and APC-CD206 (1 : 200) were used for intracellular staining. Flow cytometry data were collected on NovoCyte and analyzed using NovoExpress software (ACEA Biosciences, Inc., CA, USA).

### 2.11. Sirius Red and Masson Staining for Fibrosis

Paraffin sections (4 *μ*m) were stained with Picro-Sirius red (1% Sirius red in saturated picric acid solution) for 1 hour at room temperature to analyze collagen synthesis and deposition. The sections were then washed twice with 0.5% acetic acid. The water was physically removed from the slides by vigorous shaking. After dehydration using 100% ethanol three times, the sections were cleaned with xylene and mounted in a resinous medium. Image-Pro Plus 6.1 software (Media Cybernetics, MD, USA) was used to calculate the Sirius red-positive staining proportion.

For Masson staining, pancreatic tissue slices were routinely dewaxed, hydrated, and incubated in Wiegert's solution for 5-10 minutes. They were then differentiated in acidic ethanol for 5-15 seconds, slightly washed with water, and blued in Masson bluing buffer for 3-5 minutes. After washing with water, the slices were incubated in Ponceau-Fuchsin solution for 5-10 minutes, washed with a weak acid solution for 1 minute, and washed with phosphomolybdic acid solution for 1-2 minutes. The slices were subsequently stained in aniline blue solution for 1-2 minutes. They were then washed with weak acid solution, dehydrated in absolute ethanol, made transparent with dimethylbenzene, and mounted with neutral resin.

### 2.12. Statistical Analysis

The unpaired-sample Student *t*-test was used to determine statistical significance, and a *P* value less than 0.05 indicated a statistically significant difference. One-way ANOVA plus Tukey post hoc test was used to determine the difference among multiple groups, and a *P* value less than 0.05 indicated a statistically significant difference. Values were expressed as mean ± standard error of mean (SPSS statistical software, version 22.0, IBM Analytics, NY, USA). Unless indicated, the results were from at least 3 independent experiments.

## 3. Results

### 3.1. MaR1 Ameliorated the Histopathological Alterations of the Pancreas in Mice with AP

**Figure 1:**
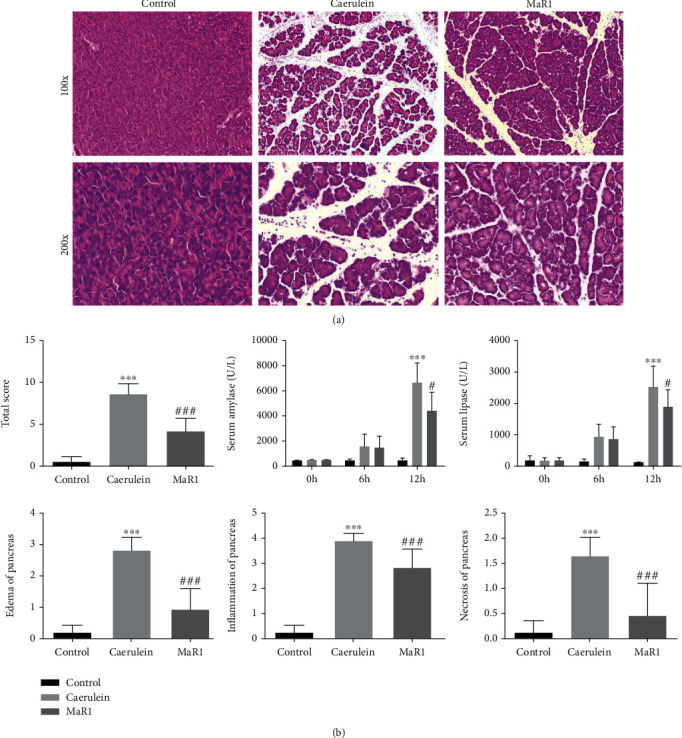
MaR1 ameliorated pancreatic tissue injury in AP mice. (a) Representative pathological changes in pancreas. HE stained sections of the pancreas in magnification 100x and 200x. (b) Histological scores of pancreatic tissues (edema, inflammation, and necrosis) and serum levels of amylase and lipase. ^∗∗∗^*P* < 0.001 vs. the control group. ^#^*P* < 0.05, ^##^*P* < 0.01, and ^###^*P* < 0.001 vs. the caerulein group. *n* ≥ 6 each group.

Our group previously reported that DHA exerted a protective effect on AP. This study investigated the effect of MaR1 (metabolite of DHA) to partly explain its clinical benefit. In animal experiments, three doses of 0.2, 2, and 10 ng/mice were adopted. As expected, the caerulein group exhibited the classical edematous pancreatitis manifestations, including edema, inflammatory cell infiltration, and spotty acinar cell necrosis. Based on the pathological results, 2 ng/mice MaR1 was chosen in subsequent experiments to examine its obvious protective effect (Figure 1(a)). Pancreatic injury scores were assessed in parallel with pathohistological changes. In addition, MaR1-treated mice also exhibited a significant reduction of serum amylase and lipase levels (Figure 1(b)).

**Figure 2:**
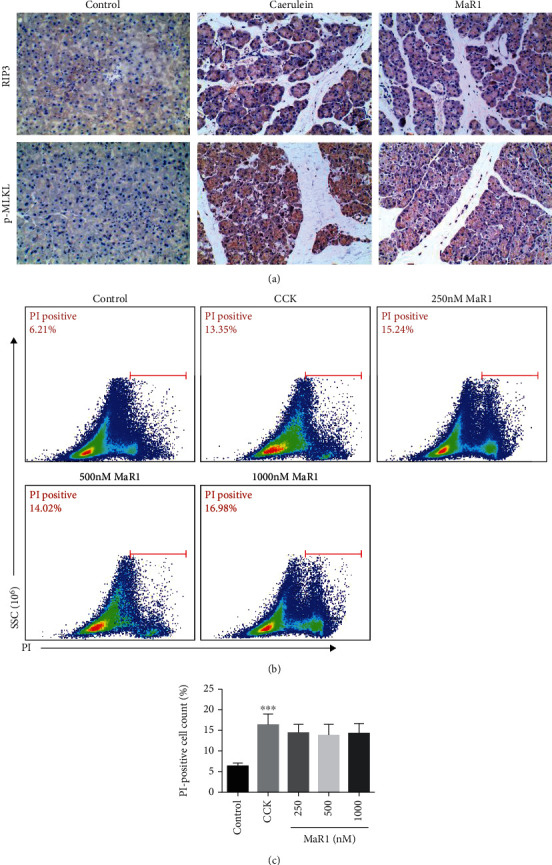
MaR1 showed protective effects independent of pancreatic acinar cells. (a) IHC examinations for RIP3 and p-MLKL of the pancreas in magnification 200x. *n* ≥ 6 each group. (b, c) Flow cytometry of PI staining for 266-6 cells and relative positive cell counting was quantified. ^∗∗∗^*P* < 0.001 vs. the control group. *n* ≥ 9 each group.

A few observations suggested that necroptosis mediated AP development. RIP kinases play a central role in regulating necroptosis [29]. RIP3 and MLKL are important proteins to assemble the necrosome. IHC examinations were used for detecting RIP3 and p-MLKL expressions in pancreatic tissues. As shown in Figure 2(a), the positive staining areas of RIP3 and p-MLKL showed a robust increase after PAC damage. MaR1 could decrease the number of RIP3- and p-MLKL-positive cells, indicating that MaR1 mitigated the severity of PAC necroptosis in the AP model.

### 3.2. MaR1 Showed No Direct Effects on Pancreatic Acinar Cell Necrosis

Based on the results of *in vivo* experiments, we first considered whether MaR1 had a direct protective effect on PAC injury. The 266-6 cell line is widely used in the pancreatitis model for its exocrine function [30, 31]. Accordingly, 266-6 cells were used to explore the effect of MaR1, and CCK was used to induce an acute injury model *in vitro*. After different doses (250 nM, 500 nM, and 1000 nM) of MaR1 treatment, PI staining demonstrated that the percent of necrotic cells in the CCK group was approximately 15.24%, while the MaR1 group showed no significant differences (Figure 2(b)). Further, the concentration gradient difference of MaR1 (50 nM, 100 nM, 250 nM, 500 nM, 750 nM, 1 *μ*M, 5 *μ*M, and 10 *μ*M) was expanded, and still no sign of protection was observed (data not shown). The results confirmed that the protective effect of MaR1 on AP might not anchor in PACs.

### 3.3. MaR1 Inhibited the Infiltration of Macrophages in the Pancreatic Tissue

**Figure 3:**
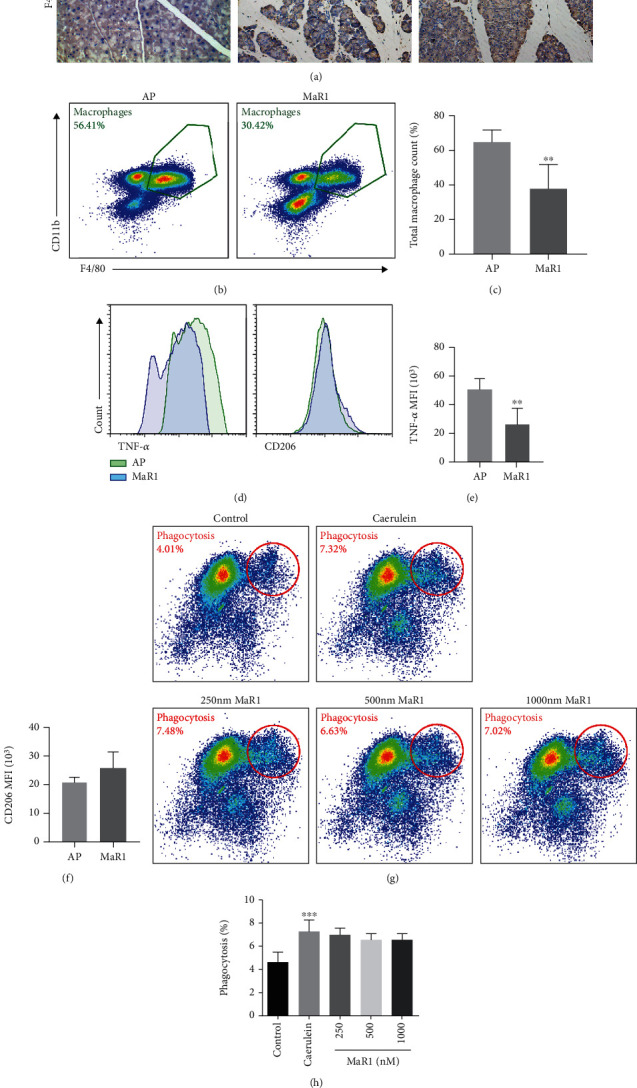
MaR1 inhibited macrophage infiltration in the pancreatic tissue. (a) IHC examinations for F4/80 of macrophages in the pancreas in magnification 200x. *n* ≥ 6 each group. (b, c) Total macrophage counting in the pancreas of the AP and MaR1 groups. (d) Flow cytometry of TNF*α* for M1 and CD206 for M2 in the pancreas. (e) Mean fluorescence intensity of TNF*α* for M1 infiltration in pancreas. (f) Mean fluorescence intensity of CD206 for M2 infiltration in the pancreas. *n* ≥ 4 each group. (g, h) Flow cytometry of BMDM phagocytosis of pancreatic acinar cells and related quantification. *n* ≥ 7 each group. ^∗∗^*P* < 0.01, ^∗∗∗^*P* < 0.001 vs. the AP or control group.

Since MaR1 was not beneficial to 266-6 cells directly, we focused on immune cells in pancreatic tissues. Macrophages play an important role in AP [32]. The IHC examination for F4/80 demonstrated that MaR1 significantly reduced macrophage infiltration in pancreatic tissues (Figure 3(a)). Primary immune cells were extracted from the pancreatic tissues of mice with AP to determine the polarization of macrophages. Flow cytometry was used to detect TNF-*α* and CD206 levels for M1- and M2-associated biomarkers, respectively. Compared to AP group, the mean fluorescence intensity (MFI) of TNF-*α* in the MaR1 group significantly reduced, while the MFI of CD206 increased slightly with no statistically significant difference (Figures 3(d)–3(f)). In addition, the total number of macrophages significantly reduced after MaR1 treatment, which was consistent with IHC staining results (Figures 3(b) and 3(c)).

MaR1 can enhance macrophage phagocytosis of dead cells and debris. We want to verify its effect in the AP model. PACs from GFP tg mice were extracted because their excitation by 488 nm light led to a fluorescence emission maximum around 530 nm. After coincubation with caerulein-stimulated PACs, BMDMs were collected for flow cytometry. The basic phagocytosis of PBS-stimulated PACs was around 4.01%. The caerulein group showed an obvious rise to 7.32%, while the MaR1 treatment group showed no change. MaR1 might not enhance BMDM phagocytosis of damaged PACs (Figures 3(g) and 3(h)). Collectively, these results indicated that MaR1 might protect against AP in mice by reducing macrophage infiltration, mainly proinflammatory M1 phenotype.

### 3.4. MaR1 Did Not Further Protect against AP after Pancreatic Macrophage Clearance

**Figure 4:**
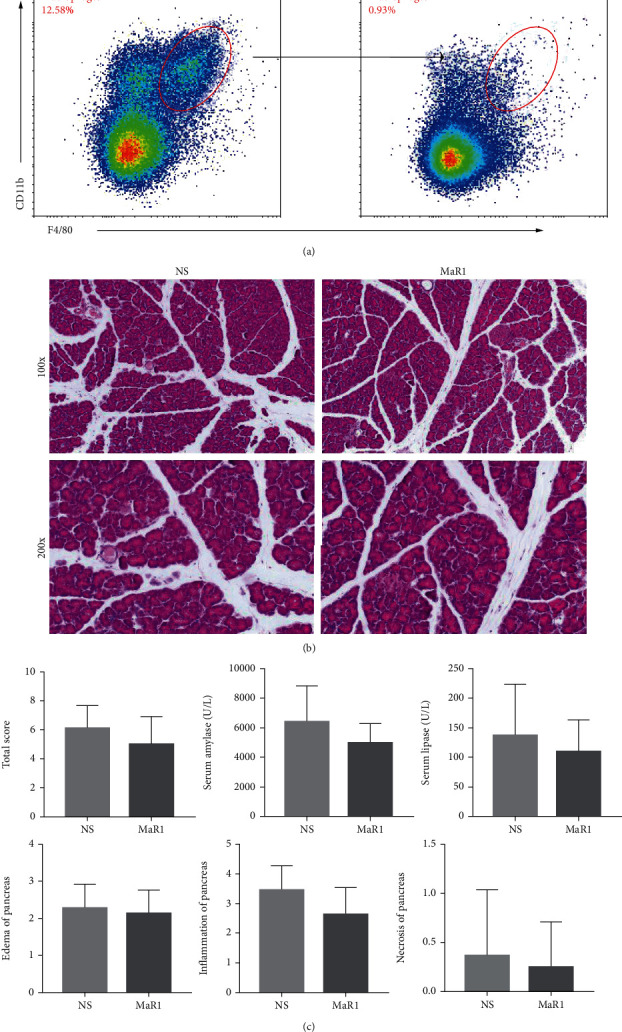
MaR1 did not further protect against AP after pancreatic macrophages clearance. (a) Flow cytometry of macrophages ratio changes in blood samples after CL injection. (b) Representative pathological changes in the pancreas after caerulein challenges. HE-stained sections of the pancreas in magnification 100x and 200x. (c) Histological scores of pancreatic tissues (edema, inflammation, and necrosis) and serum levels of amylase and lipase. *n* = 7 each group.

The pancreatic macrophages were depleted using CLs before caerulein exposure. CLs could effectively clear macrophages and protect against AP in mice (Figure 4(a)). Furthermore, MaR1 could not further alleviate the severity of experimental AP, irrespective of pathological scores or serological tests (Figures 4(b) and 4(c)). The results from both animal and cell experiments suggested that MaR1 might improve the AP severity of mice depending on pancreatic macrophages.

### 3.5. MaR1 Alleviated Macrophage Infiltration and Fibrosis of Pancreatic Tissues in the CP Model

**Figure 5:**
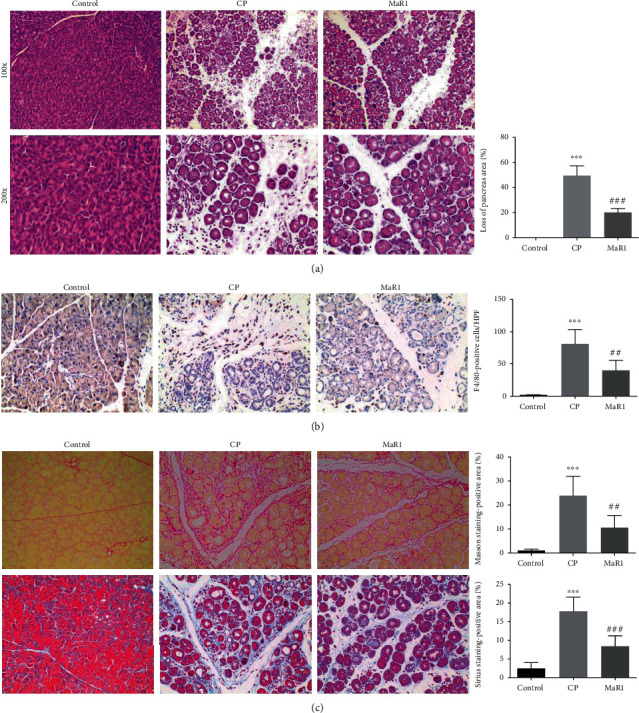
MaR1 alleviated pancreatic tissue injury and fibrosis of pancreatic tissues in CP model. (a) Representative pathological changes in the pancreas after caerulein repetitive injection. HE-stained sections of the pancreas in magnification 100x and 200x and loss of pancreas area was quantified. (b) IHC examinations and total count for F4/80 of macrophages in the pancreas in magnification 200x. (c) Masson (the upper row) and Sirius red (the lower row) staining for collagen production in the pancreas of mice. *n* ≥ 10 each group. ^∗∗∗^*P* < 0.001 vs. the control group. ^##^*P* < 0.01, ^###^*P* < 0.001 vs. the CP group.

In mice, the hyperstimulation of the pancreas with caerulein led to AP. Continuous acute injury to the pancreas caused recurrent AP and finally CP, which was consistent with the pathophysiological process of human pancreatitis. The CP model was established in a repetitive manner to further examine the effect of MaR1 *in vivo*. The mice undergoing repetitive caerulein injection revealed macrophage infiltration, pancreatic fibrosis, and acinar cell loss. Based on previous results, 2 ng/mice MaR1 was chosen, which obviously alleviated the severity of pancreatic damage (Figure 5(a)). The Sirius red and Masson staining showed that the fibrosis in the pancreas was obviously reduced (Figure 5(c)). In addition, the MaR1 group showed less macrophage infiltration (F4/80 staining) in pancreatic tissues compared with the CP group (Figure 5(b)).

## 4. Discussion

Macrophages are responsible for host defense, acute inflammatory response, and its timely resolution [33]. They can be simply divided into two extreme phenotypes: classically activated macrophages (M1) and alternatively activated macrophages (M2). Briefly, M1 macrophages exhibit proinflammation and immunologic defense properties, while M2 macrophages exhibit opposite properties. The switch from M1 to M2 phenotype can alleviate the severity of acute inflammation [34].

Since no specific treatment exists that targets pancreatic parenchyma cells, more researchers focus on local immune cells especially macrophages. Macrophages sense acinar cell death and activate pancreatic inflammation and determine the severity of AP [35]. Macrophages engulfing damaged acinar cells can also be the focus of AP in conjunction with damaged acinar cells [28]. In this study, we found that MaR1 protected AP not by acting directly on PACs but through macrophages. In accordance with Wu et al., we found that macrophage quantification in the pancreatic tissue significantly increased after AP onset (1 day) [36]. After MaR1 treatment, the percentage of macrophage population declined markedly. Furthermore, the number of M1 macrophages decreased and the number of M2 macrophages slightly increased in pancreatic tissues. Macrophages may demonstrate the plasticity and pluripotency in response to local microenvironment signals in a specific time and space. M2 macrophages may be further subdivided into M2a, M2b, M2c, and M2d, of which the surface markers are very different [37]. CD206 may not be the best choice for tracking the changes of M2. Another problem is the timing of administration of MaR1 in mice. In the tibial fracture injury model, MaR1 treatment at the time of injury is ineffective in decreasing the number of proinflammatory macrophages, different from the treatment results after injury [38]. Macrophages exhibit dynamic transitions in phenotype and function as AP progresses. The number of M2 or M2-like macrophages increases after the acute inflammation stage [36]. Delayed administration for MaR1 may provide some hints about its effect on M2 macrophages. Further, like our results, some studies reported that MaR1 markedly decreased the number of proinflammatory macrophages, but not that of anti-inflammatory macrophages in inflammation models [38, 39]. In addition, MaR1 can enhance macrophage phagocytosis of neutrophils during inflammation. However, no reports mentioned the effect of MaR1 on macrophage phagocytosis of damaged acinar cells in AP. In our study, no enhancement effect of MaR1 on the phagocytosis of injured acinar cells was observed. The results indicated that MaR1 mainly regulated macrophage phenotype to mitigate inflammation. Moreover, the findings indicated that MaR1 indeed had no further protective effect on AP after macrophage clearance in the animal model. However, keeping macrophage polarization in balance is an attractive therapeutic option for AP, considering the heterogeneous function of macrophages in different stages of diseases, besides directly eliminating macrophages.

The recurrence of AP is a challenge in clinical treatment. The incidence of recurrent AP can reach 21%, and CP develops in 36% of patients [40]. Further, effective preventive and therapeutic strategies for CP treatment are still lacking. Macrophage infiltration and activation play an important role in pancreatic injury and later fibrosis. Therefore, the CP model was used in this study to explore the pharmacological action of MaR1. MaR1 obviously attenuated macrophage infiltration, fibrosis, and pancreatic damage in the CP model. These results showed that MaR1 might serve as an immune resolvent for the clinical prevention of CP.

In conclusion, our study showed that MaR1 could decrease the severity of AP via reducing macrophage infiltration, especially M1 macrophages in pancreatic tissues. This provided evidence for the protective effect of DHA against AP. Hence, MaR1 may serve as a promising clinical therapeutic drug for treating AP in the future.

## Data Availability

All data included in this study are available upon request by contact with the corresponding author. Correspondence should be addressed to Weiqin Li, njzy_pancrea@163.com or Zhihui Tong, njzyantol@hotmail.com.
